# Intestinal microbiota profiles of captive-bred cynomolgus macaques reveal influence of biogeography and age

**DOI:** 10.1186/s42523-025-00409-9

**Published:** 2025-05-14

**Authors:** C. Purse, A. Parker, S. A. James, D. J. Baker, C. J. Moss, R. Evans, J. Durham, S. G. P. Funnell, S. R. Carding

**Affiliations:** 1 Food, Microbiome and Health, Quadram Institute Bioscience, Norwich Research Park, Norwich, NR4 7UQ UK; 2 UK Health Security Agency, Porton Down, Salisbury, SP4 0JG UK; 3 Norwich Medical School, University of East Anglia, Norwich, NR4 7TJ UK

**Keywords:** Non-human primate, Cynomolgus macaque, Microbiome, Ageing, Intestinal biogeography, Metagenomics, Reference-based computational profiling, Assembly-based computational profiling.

## Abstract

**Background:**

Age-associated changes to the intestinal microbiome may be linked to inflammageing and the development of age-related chronic diseases. Cynomolgus macaques, a common animal model in biomedical research, have strong genetic physiological similarities to humans and may serve as beneficial models for the effect of age on the human microbiome. However, age-associated changes to their intestinal microbiome have previously only been investigated in faecal samples. Here, we have characterised and investigated the effects of age in the cynomolgus macaque intestinal tract in luminal samples from both the small and large intestine.

**Results:**

Whole metagenomic shotgun sequencing was used to analyse the microbial communities in intestinal content obtained from six different intestinal regions*,* covering the duodenum to distal colon*,* of 24 healthy, captive-bred cynomolgus macaques, ranging in age from 4 to 20 years. Both reference-based and assembly-based computational profiling approaches were used to analyse changes to intestinal microbiota composition and metabolic potential associated with intestinal biogeography and age. Reference-based computational profiling revealed a significant and progressive increase in both species richness and evenness along the intestinal tract. The microbial community composition also significantly differed between the small intestine, caecum, and colon. Notably, no significant changes in the taxonomic abundance of individual taxa with age were found except when sex was included as a covariate. Additionally, using an assembly-based computational profiling approach, 156 putative novel bacterial and archaeal species were identified.

**Conclusions:**

We observed limited effects of age on the composition of the luminal microbiota in the profiled regions of the intestinal tract except when sex was included as a covariate. The enteric microbial communities of the small and the large intestine were, however, distinct, highlighting the limitations of frequently used faecal microbial profiling as a proxy for the intestinal microbiota. The identification of a number of putative novel microbial taxa contributes to knowledge of the full diversity of the cynomolgus macaque intestinal microbiome.

**Supplementary Information:**

The online version contains supplementary material available at 10.1186/s42523-025-00409-9.

## Background

The intestinal microbiota contains a diverse community of bacteria, fungi, archaea, and viruses [1]. These microorganisms ordinarily maintain a symbiotic relationship with the host organism, with numerous microbiota-derived metabolites having a beneficial effect on maintaining immune homeostasis, either through regulation of intestinal barrier integrity or immunomodulatory effects [2–15]. However, several factors can influence the composition of the intestinal microbiota, including medications, diet, behaviour, and age [16–20]. An altered intestinal microbiota composition, referred to as intestinal dysbiosis, may impair immune responses and is linked to the pathogenesis of several human chronic diseases [19, 21–25].

Previous studies in humans have demonstrated that the taxonomic composition of the intestinal microbiota is influenced by advancing age, linking this to the hypothesis that age-associated intestinal dysbiosis may underlie the development of age-associated inflammation (inflammageing) and chronic diseases [19, 26, 27]. To study the impact of intestinal dysbiosis on the ageing process, however, suitable animal models need to be identified in which intestinal dysbiosis naturally develops with age, similarly to humans. Non-human primate (NHP) species such as cynomolgus macaques (*Macaca fascicularis*), an Old-World NHP species commonly used in biomedical research, are genetically, physiologically, and behaviourally more like humans than rodent models. In captivity, their outbred nature and less tightly controlled environmental conditions also allow for validation of findings made using genetically inbred rodent models [28, 29].

The intestinal microbiota of cynomolgus macaques has previously been reported to alter with age, potentially making them an appropriate model for studying age-associated intestinal dysbiosis in humans [30, 31]. However, all previous studies of the effect of age on the intestinal microbiome in captive cynomolgus macaques have utilised 16S ribosomal RNA (rRNA) analysis of the excreted or rectally sampled faecal microbiota as a surrogate of intestinal microbial communities [30–32]. The limited taxonomic resolution of marker-based sequencing approaches, in addition to the poor representation of intestinal microbiota composition by faecal samples, restricts the understanding of how age affects the intestinal microbiota in these important animal models [33–35].

Reliance on faecal samples to profile the intestinal microbiome has resulted in limited understanding of intestinal biogeography. Investigation of intestinal biogeography in cynomolgus macaques has not previously been attempted, although one study exists in captive rhesus macaques [36]. The study examined microbial community composition in different spatial regions in the small and the large intestine, as well as differences in taxonomy and function between the mucosal and luminal microbial communities, significantly advancing knowledge of the intestinal microbiome in rhesus macaques. However, luminal samples were only obtained from the ileum onwards, providing limited insights into the small intestinal microbiota.

Previous studies that have applied shotgun-based metagenomics to NHP microbiomes used a reference-based computational profiling approach [37–40]. These studies were limited in their ability to characterise a significant proportion of these datasets due to the scarcity of NHP microbiome-specific microbial genomes within the associated reference databases [41]. A later, assembly-based, analysis expanded the catalogue of microbial genomes associated with NHP microbiomes and integrated them into the database of a commonly used reference-based computational profiling tool [41, 42]. This offers the potential for improved characterisation of the cynomolgus macaque intestinal microbiome using reference-based computational profiling methods. Despite this, the use of assembly-based approaches alongside reference-based approaches remains beneficial, especially for rarely characterised microbial communities, as it allows for identification of microbial genomes not included within reference databases, as well as novel microbial genomes.

To better understand the intestinal microbiota in cynomolgus macaques, we undertook a survey of the intestinal prokaryome, from duodenum to distal colon, in captive-bred animals aged 4–20 years using shotgun sequencing methods. In addition, by combining reference- and assembly-based approaches, we examined bacterial and archaeal diversity, the impact of age on microbial composition, and the metabolic potential of the microbiome. Our goal was to gain a more detailed insight into the taxonomic diversity and metabolic potential of the intestinal microbiome in captive-bred cynomolgus macaques and identify how age effects these microbial communities.

## Materials and methods

### Non-human primates

All animal procedures were approved by the UK Health Security Agency (UKHSA) Porton Down Establishment Animal Welfare and Ethical Review Body (Project License: PD28B8ED5) and authorised under a UK Home Office license to breed, supply, and use macaques for scientific research (Establishment license: XBF9440B0). All animals were housed and captive-bred at a UKHSA facility and derived from Mauritian or South-East Asian origin. No new animals have been introduced to these colonies since 2004. The breeding colonies were, and continue to be, maintained to the highest standard in terms of animal welfare, health status, genetic profile, and behavioural compatibility, compliant with the UK Home Office Code of Practice for the Housing, and Care of Animals Bred, Supplied or Used for Scientific Purposes, 2014. Animals were housed in compatible social groups, either in harem breeding groups, or single sex, aged-matched holding groups. Their accommodation consisted of climate controlled, multiple room, solid floor caging systems, with a floor of deep litter in the largest rooms to allow foraging and access to a non-climate controlled external ‘extension’ pen which is open to the elements. From January 2021, the climate control conditions were as follows: temperature 23 °C ± 2 °C; relative humidity 55% ± 10%; 10 air changes per hour, 12:12 light–dark cycle. Prior to January 2021 the temperature was maintained at approximately 21°C with 10 air changes per hour and ambient humidity. Additional complex enrichment was provided to fulfil the behavioural needs of the animals. Water and complete primate diet (2050/2050A, Teklad Global Diets®, Teklad™, Indianapolis, Indiana, USA) was provided ad libitum, and supplemented daily with fruits, vegetables, and pulses. None of the animals included in the present study were used previously for experimental procedures. All animals used in this study were required to be euthanised as part of colony management requirements.

### Analysed datasets

Animals included within this study were culled for reasons separate to the study objectives, such as ex-breeder status designation or diagnosis of non-infectious disease or illness. 111 luminal samples from six regions of the intestinal tract obtained from 24 cynomolgus macaques (19 females, 5 males), ranging in age from 4–20 years, were collected on an ad hoc basis upon autopsy. Only healthy animals or animals which had been culled within a short time-period of being diagnosed with diabetes, for which no treatment was administered, were included within this study. It was not possible to collect samples from all six regions from all animals, resulting in variable sample numbers per region studied. All samples were processed and sequenced as described in the following sections. Following read trimming and filtering, only samples with > 1 million reads were retained for further analysis (a threshold previously determined to be sufficient for achieving < 1% dissimilarity to the full taxonomic composition of metagenomic samples [43]). The resulting dataset contained samples from 24 cynomolgus macaques, ranging in age from 4–20 years (19 females, 5 males) (Table 1). Where necessary for analysis, animals were categorised into three age groups, young (≤ 7 years;* n* = 4), adult (8–12 years; *n* = 4), and aged (≥ 13 years; *n* = 16).

**Table 1:** Overview of total number of samples included in the present study

	Number of Samples			
Region	Young	Adult	Aged	Total
Duodenum	1	1	2	4
Jejunum	1	1	6	8
Ileum	2	2	7	11
Caecum	3	3	14	20
PC	2	2	13	17
DC	3	0	14	17

*PC* proximal colon, *DC* distal colon

### Sample collection

Luminal content samples from six regions of the intestinal tract (duodenum, jejunum, ileum, caecum, proximal colon, distal colon) were collected upon autopsy and snap frozen on dry ice. All samples were stored at −70ºC prior to processing.

### DNA extraction

Total microbial DNA was extracted from approximately 200 mg luminal content using the FastDNA™ Spin Kit for Soil DNA Extraction (MP Biomedicals, Santa Ana, California, USA), following manufacturer’s instructions. DNA was quantified and quality checked using a Qubit 3.0 fluorometer and associated Qubit dsDNA Broad Range Assay Kit (Invitrogen, Waltham, Massachusetts, USA). For each batch, a bead-beating tube containing PBS only underwent the same protocol, serving as an extraction control. DNA samples were stored at −20 ºC prior to library preparation.

### Library preparation and sequencing

DNA sample concentration was normalised to 5 ng/uL prior to library preparation. A tagmentation mix containing 0.5 µL Tagmentation Buffer 1 (TB1), 0.5 μL bead-linked transposomes (BLT) and 4.0 μL PCR-grade water was prepared and 5 µL per sample added to the well of a chilled 96-well microtitre plate. 2 µL normalised DNA (10 ng) was pipette mixed with the tagementation mix (5 μL) and heated to 55 ºC for 15 min in a PCR block. A PCR master mix was made up with 10 μL KAPA2G Fast Hot Start Ready Mix (Merck, Rahway, New Jersey, USA) and 2 μL PCR-grade water per sample. 12 μL of the master mix was combined with 1 μL of 10 μM 8 bp Unique Dual Indexes (Illumina, San Diego, California, USA) and 7 μL of tagmentation mix and PCR-amplified with the following cycling parameters: 72 ⁰C for 3 min, 95 ⁰C for 1 min, 14 cycles of 95 ⁰C for 10 s, 55 ⁰C for 20 s and 72 ⁰C for 3 min. Libraries were quantified using the Quantifluor® dsDNA system and a GloMax® Discover Microplate Reader (Promega, Madison, Wisconsin, USA). Libraries were pooled following quantification in equal quantities. The final pool was double-SPRI size selected between 0.5 and 0.7X bead volumes using sample purification beads (Illumina® DNA Prep). The final pool was quantified on a Qubit 3.0 instrument and run using the D5000 ScreenTape Assay (Agilent, Santa Clara, California, USA) on the Agilent Tapestation 4200 to calculate the final library pool molarity. Extraction controls were included within the library preparation procedure but failed due to low input of genomic material, suggesting no or minimal DNA contamination. Pools were sent to Source BioScience (Nottingham, UK) and run using the NovaSeq 6000 or NovaSeq X system.

### Taxonomic and functional characterisation

Raw sequence reads were trimmed to a minimum quality score of phred 30 and adapter sequences were removed using Fastp (v0.23.1) [44]. Host reads were determined and removed by mapping reads to the cynomolgus macaque genome (Macaca fascicularis T2T-MFA8v1.0) using BBmap from BBtools (v36.79) (sourceforge.net/projects/bbmap). Calculation of raw sequence reads following host read removal was carried out using SeqFu (v1.17) [45]. Per-sample read numbers are shown in Additional File 1.

Taxonomic profiling was performed on filtered reads using MetaPhlAn (v4.1) with the database mpa_vJun23_CHOCOPhlAnSGB_202307 [42, 46]. The size of the unknown fraction was estimated using the parameter: $--unclassified\_estimation$. Assignment of reads to MetaCyc pathways was performed using HUMAnN3 (v3.9) with DIAMOND (v2.1.9) and MetaPhlAn (v4.1), and using the mpa_vJun23_CHOCOPhlAnSGB_202307 and uniref90 (v201901b) databases [42, 46, 47]. Taxonomic and functional read abundances were normalised using copies per million (CoPM).

### Genome reconstruction and clustering

Metagenome assembled genomes (MAGs) were reconstructed from trimmed and decontaminated reads using MetaWRAP (v1.2.1) [48]. Single sample assembly was carried out using MEGAHIT (v1.2.9) [49]. Summary statistics of the assembly were generated using QUAST [50]. Contigs longer than 1000 nucleotides were filtered using seqtk (v1.4.0) [51] and binned using MetaBAT2 (v2.15) [52], Maxbin2 (v2.2.6) [53], and Concoct (v1.1.0) [54] and consolidated using MetaWRAP’s bin_refinement module, with completion and contamination parameters set as 50% completion and 10% contamination. Bins were dereplicated using dRep (v3.4.3) [55], yielding 470 medium quality MAGs (completion > 50% and contamination < 10%) and 258 high quality MAGs (completion > 90% and contamination < 5%), as assessed by CheckM [56]. MAGs were clustered at 5% genetic distance using dRep, resulting in 728 species-level genome bins (SGBs). Taxonomy was assigned using the Genome Taxonomy Database Tool Kit (GTDB-Tk) (v2.4.0) and GTDB release 220 [57–60]. 156 SGBs were undefined at species level, representing putative novel taxa. Abundance of each putative novel SGB was quantified using MetaWRAPs quant_bin module. Abundance was normalised to copies per million (CPM). Data visualisation was carried out in R (v4.3.2) using RStudio (v2024.04.2) [61, 62]. The scripts used for data visualisation and analysis are available from the Github repository https://github.com/Cat-Elin/1224_CMBiogeographyManuscript (December 2024).

### Alpha and beta diversity analyses

Species relative abundances obtained from MetaPhlAn (v4.1) were imported into R and manipulated using the *phyloseq* package (v1.48.0) [63]. Alpha diversity metrics (Chao1, Inverse Simpson’s index, and Shannon diversity) were calculated using the alpha function from the microbiome package (v1.24.0) [64]. To facilitate statistical comparisons of alpha diversity values between intestinal regions a linear mixed effects model was created using the nlme package (v3.1.163) [65]. The model specification was as follows:

$$lme\left(diversity \sim type+age, random=\sim 1|ID, data=data, method={\text{REML}}\right).$$

“ID” refers to each animal’s unique identification code, “diversity” refers to the relevant alpha diversity metric, and “data” refers to the relevant dataset. ID was included as a random effect (~ 1|ID) to account for intra-subject correlations in diversity measurements obtained from intestinal regions within the same animal. The intestinal region (“type”) was included as a fixed effect to assess the relationship between intestinal region and alpha diversity. “Age” was also included as a fixed effect as it is a potential confounding factor. The model is fit by maximising the restricted log-likelihood (“REML”). The Benjamini-Hochberg (BH) procedure was applied to the raw p-values to control for multiple comparisons and adjust for the false discovery rate (FDR).

To facilitate comparisons of alpha diversity values with age per intestinal region, linear models with the following model specification were created using the stats (v4.3.2) package [61]:

$$lm(diversity \sim age, data=data)$$

where “diversity” refers to the relevant alpha diversity metric, “age” refers to continuous age, and “data” refers to the relevant dataset.

To assess beta diversity, a non-metric multidimensional scaling (NMDS) ordination based on Bray–Curtis dissimilarities was performed using the *phyloseq* package. Statistical differences between age groups and intestinal regions were assessed by performing a permutational multivariate analysis of variance (PERMANOVA) with 999 permutations using the function *Adonis2* from the R package *Vegan* (v2.6.6.1) [66]. Permutations were constrained within samples obtained from the same animal to account for intra-subject correlations in diversity measurements.

All statistical analysis testing associations of alpha and beta diversity with intestinal region and age were also run with sex included as a covariate. The results of this analysis are in Additional Files 11 – 13.

### Analysis of associations between taxonomic and functional features and age

To assess significant associations between the relative abundance of both characterised and putative novel prokaryotic taxa and MetaCyc pathways with increasing age, the abundance of taxa and functional pathways per intestinal region were analysed using MaAsLin2 [67]. Continuous age was set as a fixed effect. An FDR of 10% was used. Each analysis was performed twice, once without and once with sex included as a fixed effect. For each analysis, the following parameters were used: $min\_prevalence = 0.4, max\_significance = 0.1, normalisation = "NONE", transform = "none", analysis\_method = "LM", correction= "BH"$.

## Results

To investigate the effect of age and biogeography on the intestinal microbiome in captive-bred cynomolgus macaques, 77 luminal content samples from 6 regions (duodenum to distal colon) of the intestinal tract from 24 cynomolgus macaques of differing ages (4–20 years) were sequenced. An overview of the samples and the associated metadata is in Additional File 3.

**Fig. 1:**
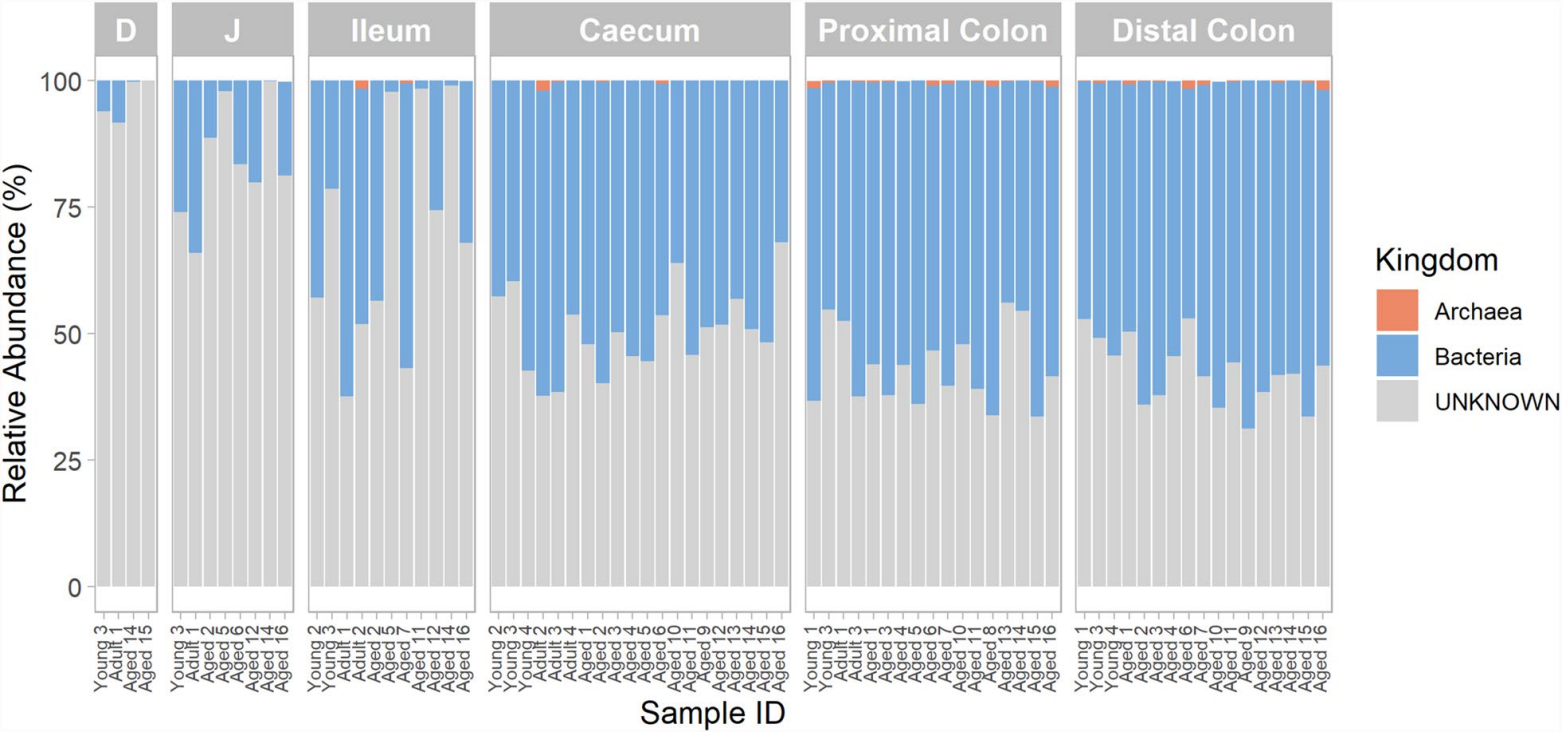
Kingdom-level intestinal microbiota composition across different spatial regions in captive cynomolgus macaques. Age groups are defined as 4–7 years (Young), 8–12 years (Adult) and 13–20 years (Aged). Within each facet, individual bars represent samples from different animals, arranged in order of ascending age from left to right. *D* Duodenum, *J* Jejunum

To investigate the taxonomic composition of the microbiota in different spatial regions of the intestinal tract, we initially employed a reference-based approach. On average, approximately 55.36% (± 19.70) of the estimated total number of microbial genomes could be characterised using this approach (Fig. 1). Archaea were identified in 39 samples, with an average relative abundance of 0.25% (± 0.46). The highest percentage of archaeal taxa in any sample was 1.96%.

**Fig. 2:**
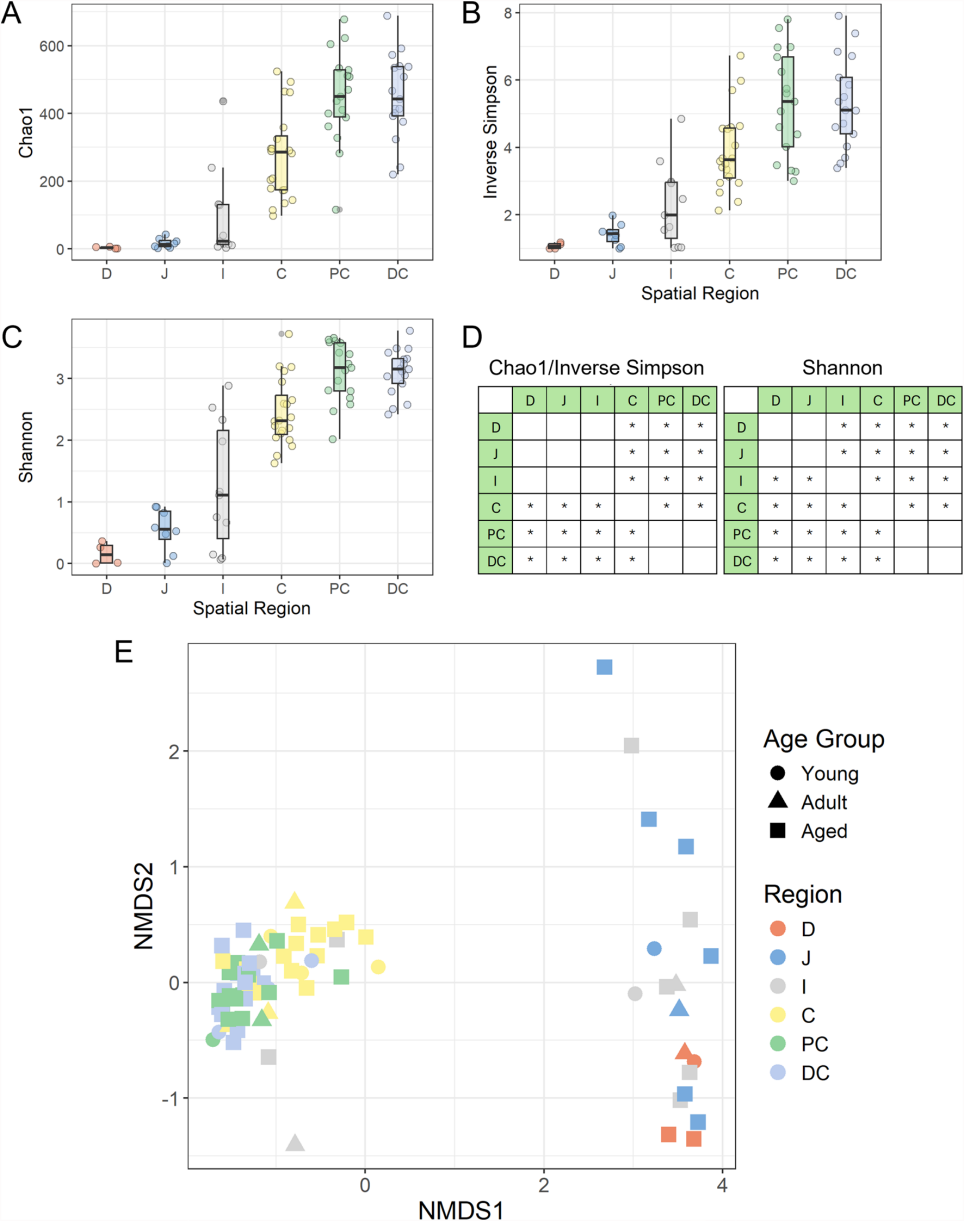
Diversity of intestinal microbiota composition across regions in captive cynomolgus macaques of differing ages. Alpha diversity is assessed using three metrics: **A** Chao1 index **B** Inverse Simpson’s index **C** Shannon’s index. **D** Tables summarizing statistically significant differences in alpha diversity between intestinal regions (* = p < 0.05). **E** NMDS plot based on Bray–Curtis dissimilarity, showing the clustering of samples by intestinal region and age group. Age groups are defined as 4–7 years (Young), 8–12 years (Adult) and 13–20 years (Aged). *D* Duodenum, *J* Jejunum, *I* Ileum, *C* Caecum, *PC* Proximal Colon, *DC* Distal Colon

Alpha diversity metrics were used to compare species richness and evenness across intestinal spatial regions (Fig. 2A-D). Age did not demonstrate a significant association with diversity when controlling for region. When comparing intestinal regions, Chao1 and Inverse Simpson’s index were observed to progressively and significantly increase in the caecum, the proximal colon, and the distal colon compared to the duodenum, jejunum, and ileum (p < 0.05) (Additional File 4). Shannon diversity progressively and significantly increased in the ileum, caecum, proximal colon, and distal colon compared to the duodenum and jejunum (p < 0.05) (Additional File 4). The same significant associations were observed when statistical models were adjusted for sex in addition to region and age (Additional File 11).

Using data obtained from the reference-based profiling approach, beta diversity at the species level was assessed using a NMDS plot based on Bray–Curtis community dissimilarities (Fig. 2E). A PERMANOVA was conducted to assess the impact of age and intestinal region on community composition, with the analysis revealing that approximately 36.56% of the total variance could be explained by these factors. Pairwise statistical comparisons revealed no differences in community composition between age groups but did reveal distinct clustering patterns across different intestinal regions (Additional File 5). The microbial communities of the duodenum, jejunum, and ileum exhibited similarities and formed a distinct cluster, as did the proximal and distal colon. The microbial composition of the caecum appeared distinct from all other regions. The same clustering patterns were observed if the PERMANOVA was adjusted for sex in addition to intestinal region and age, with sex estimated to account for only 1.22% of the total variance, although this was not significant (Additional File 12). These findings were suggestive that environmental factors, such as pH or nutrient availability, influenced microbial community composition in each intestinal region, although other, unknown, factors also accounted for a significant proportion of the total variance.

**Fig. 3:**
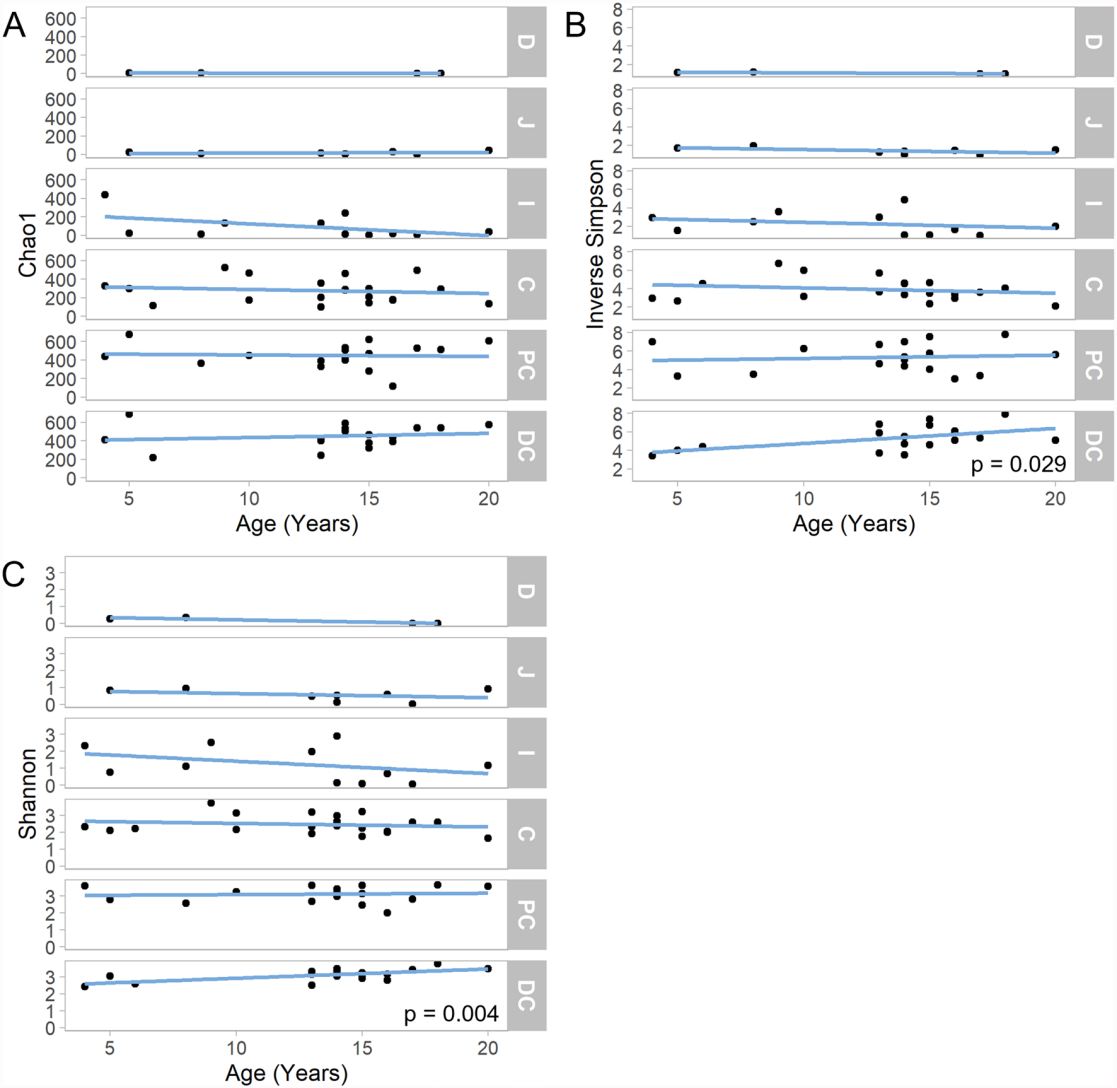
The effect of age on region-specific intestinal microbiota alpha diversity in captive cynomolgus macaques. Alpha diversity is assessed using three metrics: **A** Chao1 index **B** Inverse Simpson’s index **C** Shannon’s index. Where the correlation between age and alpha diversity reaches statistical significance (p < 0.05) the p-value is displayed on the plot. *D* Duodenum, *J* Jejunum, *I * Ileum, *C*Caecum, *PC* Proximal Colon, *DC* Distal Colon

Region-specific assessments of the association between increasing age and alpha diversity were also carried out. Three alpha diversity metrics, Chao1, Inverse Simpson’s index, and Shannon diversity, were assessed (Fig. 3A-C). Significant, positive associations between age and Shannon’s diversity (p = 0.004) and Inverse Simpson’s index (p = 0.029) were observed in the distal colon. In contrast, no significant correlation between age and Chao1 was observed, suggesting that while species evenness may increase with age in the distal colon, species richness remains stable. No significant correlation was observed between age and alpha diversity in any other intestinal region (Additional File 6). When sex was included as a covariate within this analysis, a significant, negative association was observed between age and Shannon’s diversity (p = 0.047) and Inverse Simpson’s index (p = 0.037) in the duodenum, suggestive of a link between sex, age, and diversity in this region (Additional File 13).

**Fig. 4:**
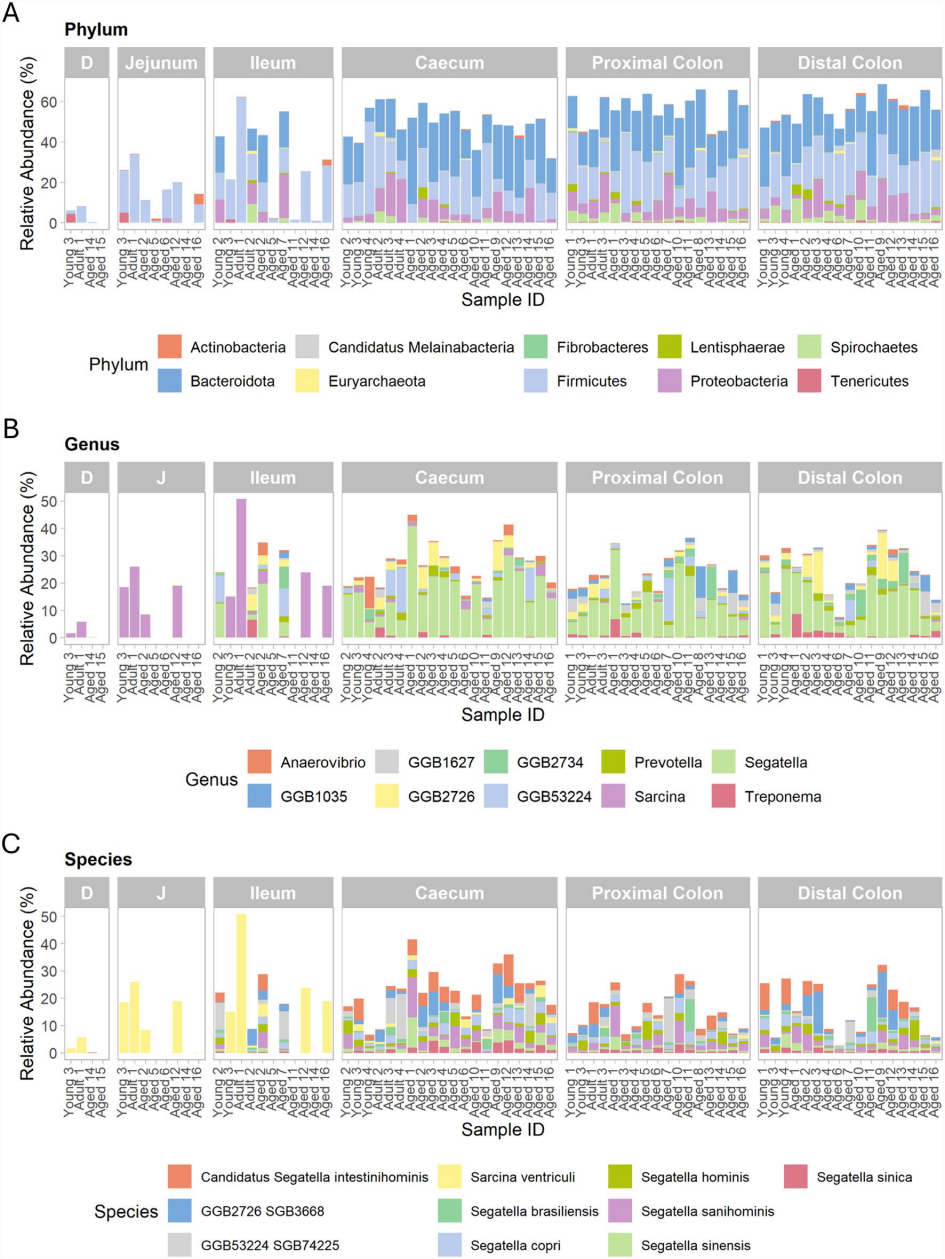
Top 10 most abundant bacterial taxa in the captive cynomolgus macaque intestinal microbiota across regions. Taxa are shown at the **A** Phylum **B** Genus and **C** Species level in animals of differing ages. Age groups are defined as 4–7 years (Young), 8–12 years (Adult) and 13–20 years (Aged). Within each facet, individual bars represent samples from different animals, arranged in order of ascending age from left to right. *D* Duodenum, *J* Jejunum

**Fig. 5:**
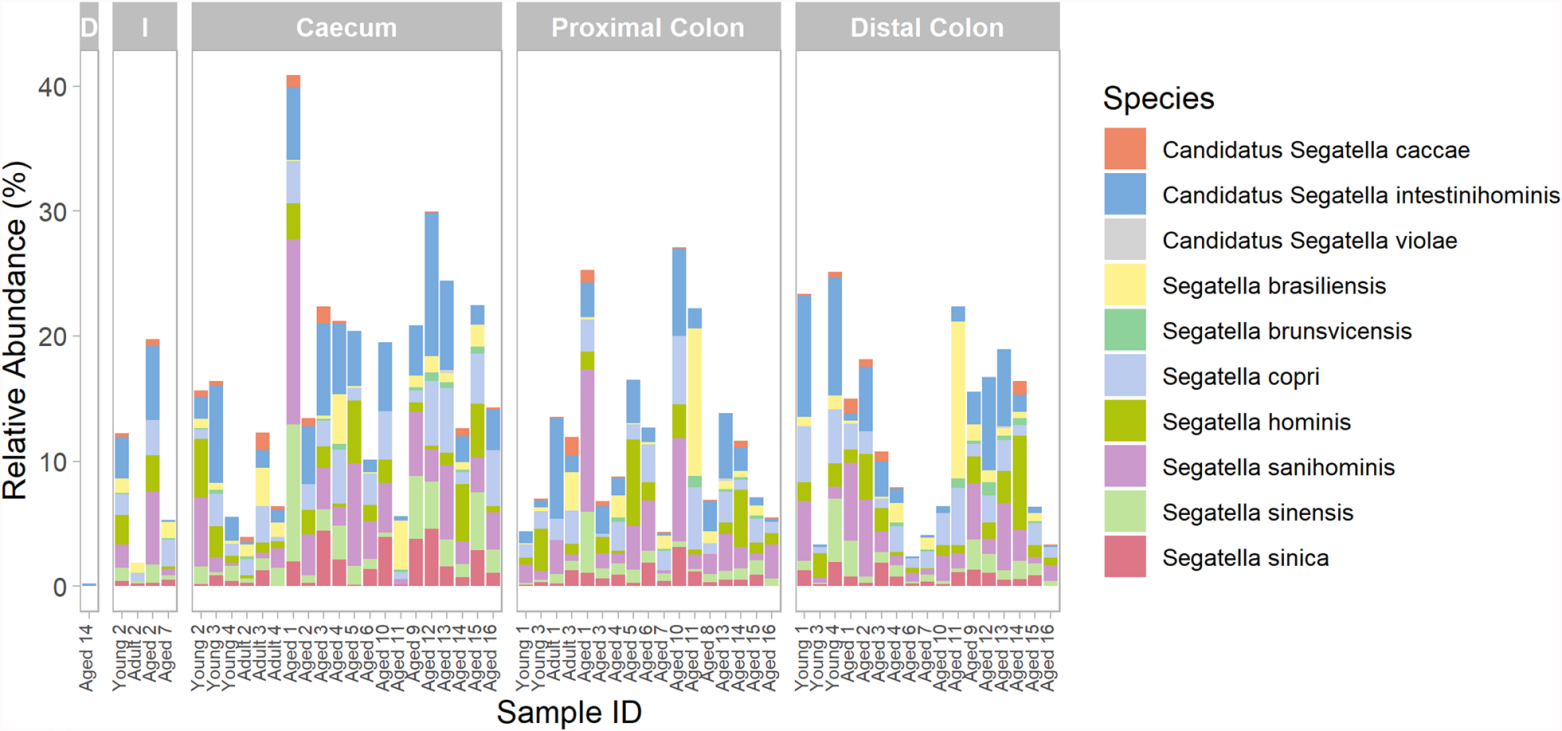
Relative abundance of *Segatella* species in the captive cynomolgus macaque intestinal microbiota across regions. Age groups are defined as 4–7 years (Young), 8–12 years (Adult) and 13–20 years (Aged). Within each facet, individual bars represent samples from different animals, arranged in order of ascending age from left to right. Only samples with greater than 0.1% relative abundance of *Segatella* are shown. *D* Duodenum, *I* Ileum

Bacterial taxonomy was assessed in samples from each intestinal region. Across both the small and large intestine, the three most abundant phyla were *Bacteroidota* (*Bacteroidetes*), *Bacillota* (*Firmicutes*), and *Pseudomonadota* (*Proteobacteria*). In the large intestine, on average, 23.5% of the identified taxa were *Bacteroidota*, 19.4% were *Bacillota*, and 8.07% were *Pseudomonadota*. In the small intestine, however, 13.8% of identified taxa were *Bacillota*, 3.1% were *Bacteroidota*, and 2.27% were *Pseudomonata* (Fig. 4A). At the genus level, the top 10 most abundant taxa included *Sarcina*, primarily in the small intestine, and *Segatella* (Fig. 4B). In small intestinal samples, the most abundant species was *Sarcina ventriculi*. Of the top 10 most abundant taxa, seven were *Segatella* or *Candidatus Segatella* species (Fig. 4C). The total number and abundance of *Segatella* species at > 0.1% relative abundance across all samples was then investigated, revealing that *Segatella* species or candidate *Segatella* species were identified in 60 out of 77 samples and in all regions except the jejunum (Fig. 5).

**Fig. 6:**
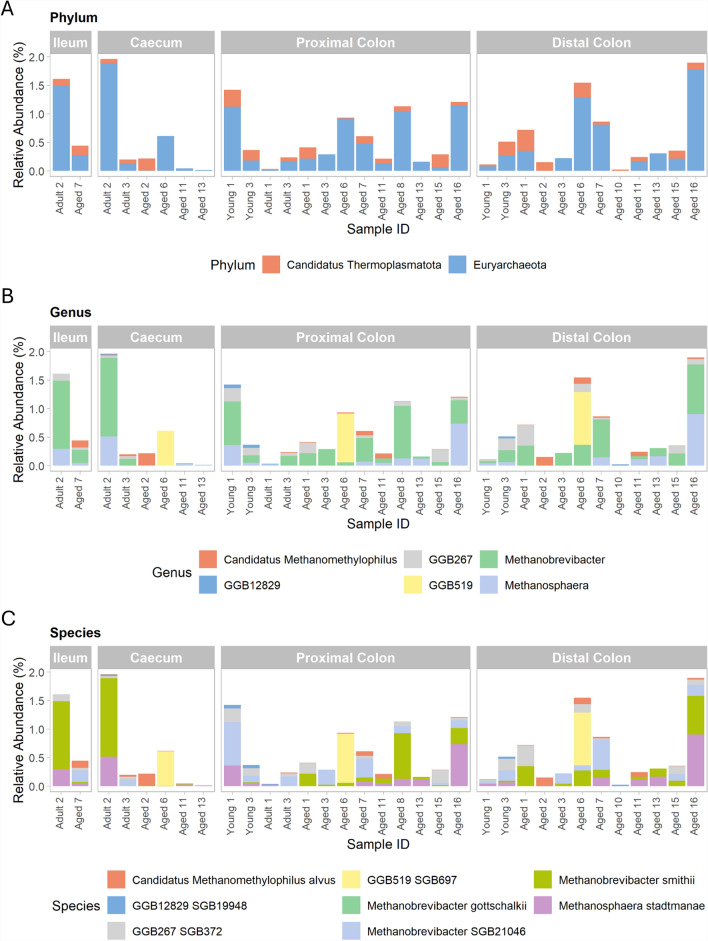
Top 10 most abundant archaeal taxa in the captive cynomolgus macaque intestinal microbiota across regions. Taxa are shown at the **A** Phylum **B** Genus and **C** Species level in animals of differing ages. Age groups are defined as 4–7 years (Young), 8–12 years (Adult) and 13–20 years (Aged). Within each facet, individual bars represent samples from different animals, arranged in order of ascending age from left to right. Only samples with greater than 0.01% relative abundance of Archaea are shown

Investigation of archaeal taxonomy revealed eight species in two phyla, *Euryarchaeota* and *Candidatus Thermoplasmatota* (Fig. 6A, 6C). Species from both phyla were identified in spatial regions from the ileum to the distal colon. *Methanobrevibacter* was the most dominant taxon at genus level (Fig. 6B).

Age associated changes in the abundance of specific bacterial or archaeal taxa was initially investigated in all intestinal regions, with no significant associations observed (Additional File 7). However, when sex was included as a covariate, the abundance of *S. ventriculi* and *Limosilactobacillus reuteri*, a species associated with probiotic properties, was negatively associated with age and male animals [68]. In the distal colon, *L. reuteri,* cellulose-degrader *Ruminococcus champanellensis*, *Ligilactobacillus animalis*, a probiotic species which has been positively associated with intestinal barrier integrity in vitro, and *Oliverpabstia intestinalis*, previously identified in the porcine intestine, in addition to a number of uncharacterised bacterial taxa, were positively associated with age [69–71]. *Methanobacteriaceae* was also positively associated with age in this region (Additional File 14).

**Fig. 7:**
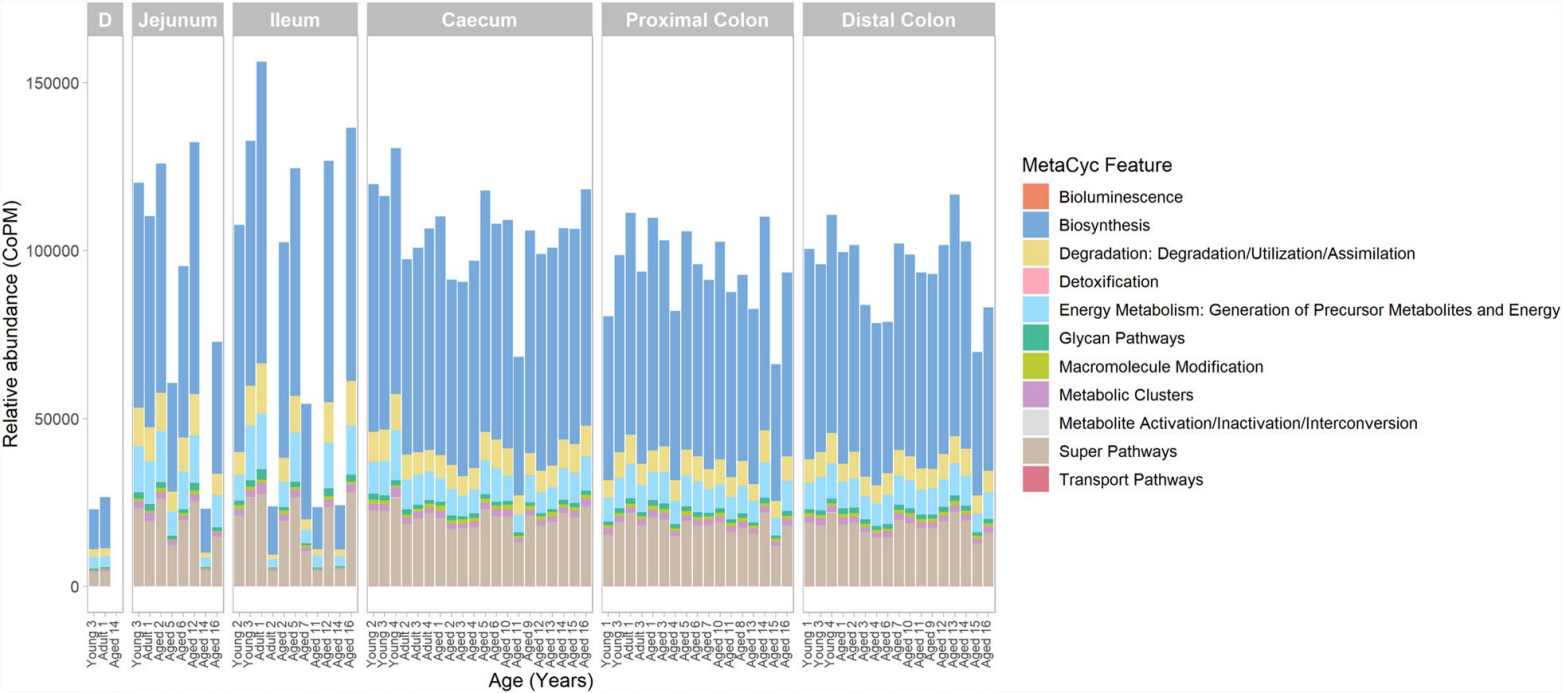
Abundance of MetaCyc pathways across different regions of the captive cynomolgus macaque intestinal tract. Pathways are categorised into higher-level organisational terms. Age groups are defined as 4–7 years (Young), 8–12 years (Adult) and 13–20 years (Aged). Within each facet, bars represent individual samples from different animals, arranged by ascending age from left to right. Pathway abundance is normalised and expressed as copies per million (CoPM) to account for variation in sequencing depth. *D *Duodenum

The metabolic potential of the intestinal microbiome was then assessed in each spatial region, with the identified pathways were sorted into broader classes of higher organisational terms for visualisation (Fig. 7). The association between the abundance of each pathway and animal age was assessed in each region, with pathway abundance found to be similar across all samples (Additional File 8). No significant changes in taxonomic composition were detected with age, and pathway abundance profiles were similar across intestinal regions when using broader pathway classifications. When sex was included as a covariate within the statistical models, however, significant associations between pathway abundance and age were observed in the ileum, proximal colon, and distal colon (Additional File 15). Most of the identified pathways in the distal colon were associated with specific taxa, such as *L. animalis*, suggesting that alterations in pathway abundance were linked to changes in taxa abundance, potentially in response to age-associated shifts in the intestinal environment.

Due to the large proportion of metagenomes that remain unclassified when carrying out reference-based taxonomic profiling, an assembly-based approach was also employed to reconstruct bacterial and archaeal genomes de novo. Following single-sample assembly and contig binning processes, 2667 MAGs were retrieved that met the criteria of completeness of > 50% and contamination of < 10%, previously defined as medium quality draft genomes [72]. Of these, 801 had completeness of > 90% and contamination of < 5%, defined as high quality draft genomes [72].

**Fig. 8:**
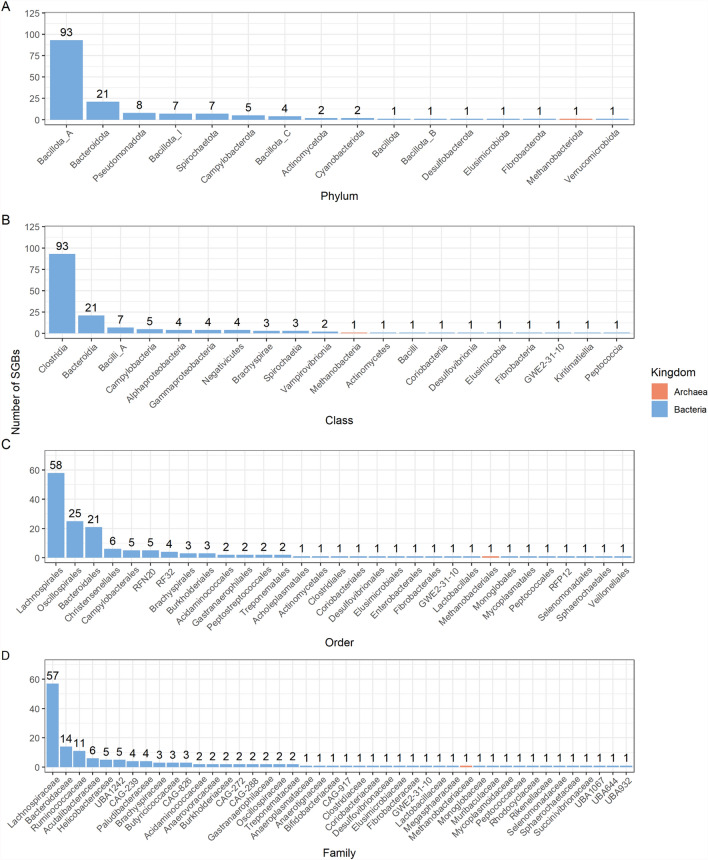
Taxonomic labels of putative novel species-level genome bins (SGBs). SGBs were obtained from the intestinal metagenomes of captive cynomolgus macaques of differing ages. Classifications are shown at the **A** Phylum level **B** Class level, **C** Order level and **D** Family level

**Fig. 9:**
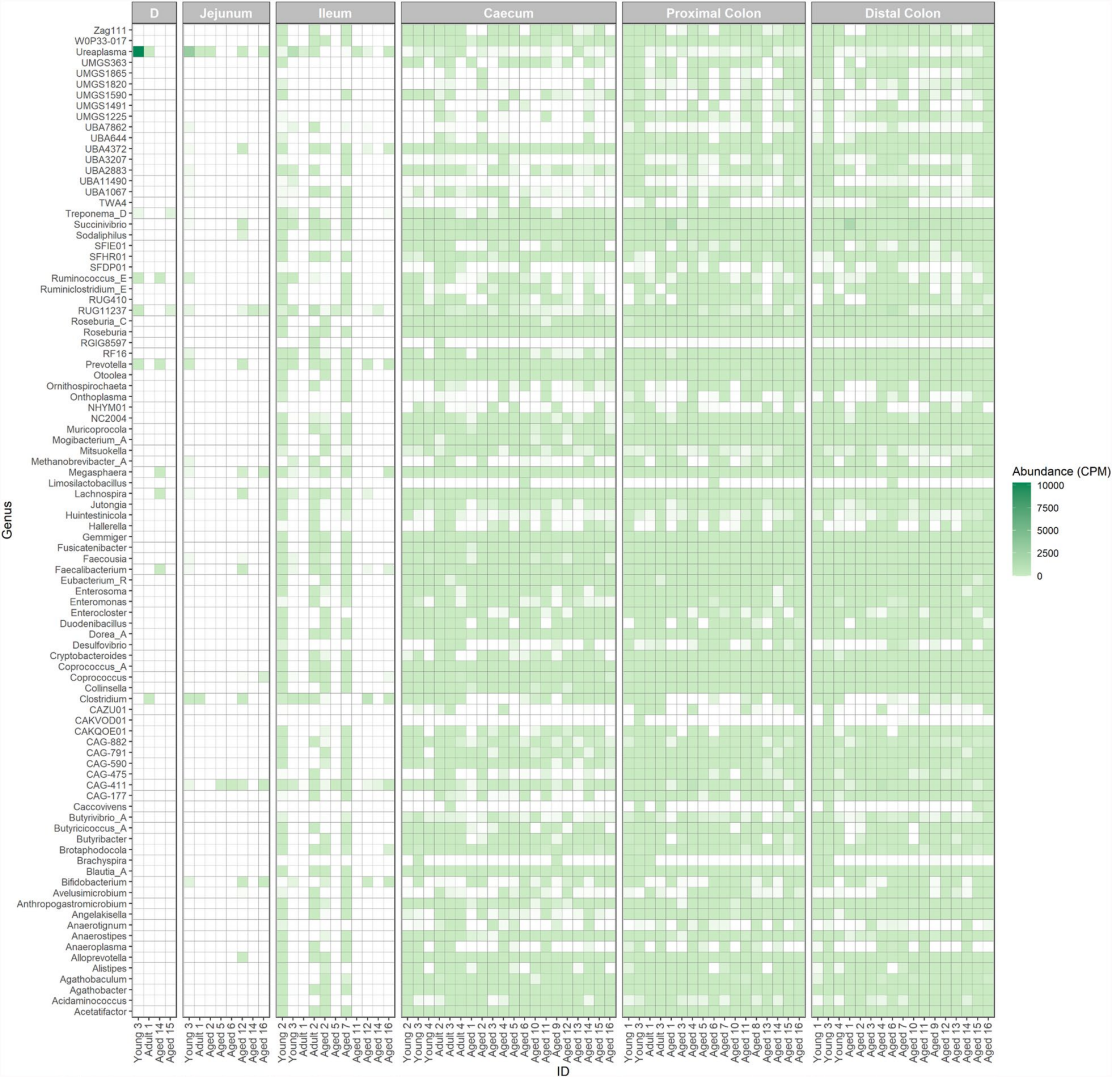
Abundance of putative novel species-level genome bins (SGBs) at the genus level. SGBs were obtained from the intestinal metagenomes of captive cynomolgus macaques across different age groups: 4–7 years (Young), 8–12 years (Adult) and 13–20 years (Aged). Within each facet, labels represent individual samples from different animals, arranged by ascending age from left to right. Abundance is normalised and expressed as counts per million (CPM), with white represents absent taxa. Abbreviations: D = Duodenum

MAGs were clustered at 95% ANI to obtain 728 SGBs, which were composed of 721 bacterial SGBs and seven archaeal SGBs. Of those not defined to species level, 155 bacterial SGBs (21.5% total SGBs) and one archaeal SGB (14.3% total SGBs) had an average nucleotide identity of < 95%, representing potentially novel species (Additional File 9). Overall, these SGBs consisted of 16 phyla, and primarily comprising bacterial taxa within the phylum *Bacillota* and class *Clostridia* (Fig. 8A, B). At order and family level, the most common taxonomic assignments were *Lachnospirales* and *Lachnospiraceae* (Fig. 8C, D). The archaeal genome was assigned to the phylum *Methanobacteriota* and genus *Methanobrevibacter* (Fig. 8A, D). The distribution of these SGBs was relatively homogenous within large intestinal regions, while largely absent from the small intestine (Fig. 9). When assessing the differential abundance of this set of putative novel SGBs with age, the abundance of two bacterial SGBs was found to correlate with age significantly and negatively in the proximal colon when the FDR was set to 10% (Additional File 2). These SGBs were assigned to the genera *UBA11490* and *RF16*. No other SGBs were found to significantly alter with age in any region (Additional File 10). When sex was included as covariate within this analysis, the abundance of five bacterial SGBs were found to positively correlate with age in the distal colon. This included the genus *RF16* and four genera within *Bacillota*; *Mitsuokella*, *Coprococcus_A*, *UMGS1865*, and *Mogibacterium_A*.

## Discussion

To investigate the effects of age on the cynomolgus macaque microbiota in multiple spatial regions of the intestinal tract, we carried out reference-based taxonomic profiling, in addition to de novo metagenomic assembly methods, to characterise both known and uncharacterised prokaryotic microbes.

### Characterisation of the captive-bred cynomolgus macaque intestinal tract using reference-based profiling

*Bacteroidota*, *Bacillota*, and *Pseudomonadota* are the most abundant phyla in all spatial regions of the intestinal microbiome in the cohort of cynomolgus macaques studied. In the large intestine the relative abundance of *Pseudomonadota* is approximately one third of that of *Bacteroidota* and *Bacillota*. This aligns with previous studies of the faecal microbiota in captive cynomolgus macaques, the human faecal microbiota, and the ileal and large intestinal luminal microbiota in captive rhesus macaques [30–32, 36, 73]. In contrast, a study of the faecal microbiota in wild rhesus macaques and wild hybrids of rhesus and cynomolgus macaques found approximately equal proportions of *Pseudomonadota*, *Bacteroidota*, and *Bacillota* in the macaque faecal microbiota [73]. This discrepancy may be related to several factors, including differences in environment, diet, and host genetics and physiology.

Species belonging to the genus *Segatella* are notably abundant within the ileum, caecum, proximal colon, and distal colon of the present NHP cohort. Further investigation of the prevalence of all *Segatella* species at > 0.1% abundance revealed the presence and identity of 10 *Segatella* species. *Segatella* was formerly part of the *Prevotella* genus [74–76]. Before the recent taxonomic revision of *Prevotella* into multiple genera, its presence was associated with the microbiomes of non-Westernised populations and those which follow plant-based, high-fibre diets, such as the Mediterranean diet [77–82]. As most of these studies could only assign taxa to genus level, it is unclear whether *Segatella* species are present within these metagenomic datasets.

An increased prevalence and diversity of *Segatella* species is associated with non-westernised compared to westernised human populations [75, 76]. Nine of the ten *Segatella* species identified within the present dataset are found mainly within human, particularly non-westernised, and captive NHP populations, while the remaining species are highly prevalent in wild, adult baboons [75]. It is possible that the prevalence of primarily human-associated *Segatella* taxa is a result of captivity. Previous studies of the faecal microbiota in wild douc langurs and howler monkeys has shown that captivity is associated with increased similarity to the human faecal microbiota [83, 84]. Housing wild-born cynomolgus macaques in captivity for one year has significant effects on the taxonomic composition of their faecal microbiota, including an enrichment of *S. copri* [83]. This study was carried out prior to the reclassification of *S. copri* into 13 distinct species, so it is unclear which of the species in the *S. copri* complex is present in this dataset. Dietary and environmental differences, including continual interaction with human handlers, in captive versus wild macaques may account for the captivity-associated enrichment of *S. copri* (complex) [83].

*S. ventriculi*, a gram-positive, anaerobic bacterium, is dominant in small intestinal samples. This bacterial species has previously been identified in several mammals, including a number of Old World NHPs, suggesting that it may be a commensal organism in the present cohort of captive cynomolgus macaques [85, 86]. In humans, it is nearly absent in meat-eaters, but is present in faecal samples of vegetarians [87]. However, in more recent studies, *S. ventriculi* is typically associated with gastrointestinal disorders, including emphysematous gastritis, gastric perforation, and gastric ulcers [88, 89]. Therefore, it is possible that *S. ventriculi* may be an opportunistic pathogen in humans, and its detection may be rare due to its localisation to the small intestine; a region that is difficult to sample and poorly characterised in humans. There are previous reports of *S. ventriculi* being associated with lethal gastrointestinal disease in NHPs [90, 91]. However, 16S rRNA sequencing has indicated that there is substantial sequence variation between strains of *S. ventriculi*, raising the possibility that virulence varies between strains [85]. Strain variation may therefore account for the absence of pathogenicity in the present cohort of cynomolgus macaques. Alternatively, tolerance for *S. ventriculi* colonisation may stem from differences in host physiology and/or microbial community interactions.

The average relative abundance of archaea in the present cohort was 0.25%, lower than previously reported in humans (approximately 1.2%) [92]. All identified archaea are methanogens from the *Euryarchaeota* and *Candidatus Thermoplasmatota* (formerly *Euryarchaeota*) phyla, aligning with findings in humans [93]. Although no studies of archaeal taxonomy and abundance exist in cynomolgus macaques, methanogens are the dominant archaeal species detected in other Old World primate species, including those belonging to *Hominidae*, and have also been identified in wild and captive rhesus macaques [36, 94, 95]. Archaeal abundance has been positively correlated with dietary fibre content in NHPs [94]. It is unclear, however, whether reduced archaeal abundance in this cohort compared to previous human studies is due to factors such as environment and diet, or limitations in the DNA extraction method used for archaea [92].

### Intestinal biogeography varies along the intestinal tract

Our findings suggest that luminal microbial community structure is distinct in the small intestine, caecum, and colon respectively. This is not unexpected, as environmental factors including intestinal pH, tissue architecture, nutrient availability, the mucus layer, and oxygen availability are known to vary along the intestinal tract, all of which may affect intestinal microbial composition [96–98]. In captive rhesus macaques, however, luminal microbial community structure does not differ between small intestinal (ileal) and colonic samples, although mucosal community structure does, possibly due to differing oxygen and nutrient availability between the lumen and the mucosa [36, 98]. The discrepancy between our findings and those in rhesus macaques may be due to differences in animal husbandry, sample collection and processing, and analysis.

Alpha diversity is also elevated in the cynomolgus macaque caecum and colon compared to the small intestine when assessed by Chao1 and Inverse Simpson’s index, similarly to previous findings in mice [99]. When assessed by Shannon’s index, alpha diversity significantly and progressively increased from the ileum onwards. However, while findings in mice suggest that there is no significant difference in alpha diversity in the caecum and the colon, our findings suggest that alpha diversity in the caecum is distinct from both the small intestine and the colon in captive cynomolgus macaques [99]. This apparent discrepancy may be due to differences in intestinal tissue anatomy in mice and cynomolgus macaques, although a study directly comparing the two is lacking. However, it is known that the anatomy of the mouse and human intestinal tract differs due to distinct dietary patterns, with the caecum known to be relatively larger in mice due to it being the primary site of fermentation, as opposed to the large intestine in humans [100]. It is possible that similar differences in intestinal anatomy exist between cynomolgus macaques and mice, contributing to differences in microbial community composition.

### There are limited age-associated changes to the captive cynomolgus macaque intestinal microbiome

Microbial community structure in different age groups is not dissimilar when assessed using beta diversity, suggesting no or limited differences. The correlation between measures of alpha diversity and age is also inconsistent across intestinal regions, with associations only reaching significance in one region whether sex is included as a covariate or not. There is also no evidence that the abundance of specific taxa or metabolic pathways significantly change with age except when sex was included as covariate. This may mean that sex is a confounder in the relationship between age and microbiome composition (potentially because of unequal age distributions between sexes in the present cohort), or that the strength of the effect of age on intestinal microbiome composition is sex dependent. The latter possibility would align with the findings of a previous study, which found that microbial community composition in anal samples obtained from wild rhesus macaques and rhesus/cynomolgus macaque hybrids was significantly associated with age in male, but not female, animals [73]. Further analysis with a larger cohort and even ratio of male and female animals would be necessary to test and provide confidence in these findings. Overall, however, our findings suggest that, while there may be changes in the proportional distribution of taxa, microbial community structure and richness is maintained with age in captive cynomolgus macaques.

The limited changes in community composition with age described here contrasts with the findings of a previous study of captive, male cynomolgus macaques [30]. The study by Wei *et*. *al*., 2022, found distinct microbial community composition profiles in faecal samples taken from different age groups, including infant (1–2 years), young adult (4–6 years), middle-aged (7–10 years), and elderly (≥ 13 years) macaques [30]. The animals were, however, individually caged, which does not reflect the social dynamics of our group-housed and wild populations of cynomolgus macaques and may have influenced the ability to discern significant results. Other factors which may lead to the discrepancy between findings include differences in cohort size, distribution of ages, methodology, and sex [30].

### Identification of putative novel microbial taxa from the cynomolgus macaque intestinal microbiome

On average, approximately half of the estimated total number of microbial genomes in the captive cynomolgus macaque intestinal microbiome could be characterised using reference-based profiling methods. As a result, we utilised an assembly-based approach to reconstruct microbial genomes de novo from our metagenomic dataset. Using a single-sample assembly approach, 156 putative novel bacterial and archaeal SGBs can be identified, all of which can be classified to genus level. Primarily, these SGBs appeared to be distributed across regions of the large intestine, from caecum to distal colon. While this may reflect differences in taxonomic composition in the small and the large intestine, it is also possible that the reduced read depth observed in small intestinal samples compared to the large intestine obscures the true distribution of these putative novel SGBs (Additional File 1).

When we tested whether the abundance of the identified SGBs was associated with age, we found that two SGBs correlated significantly and negatively, with five correlating significantly and positively with age when sex was included as a covariate. These findings highlight possible age-associated changes in taxonomy which may be obscured by the inability to characterise the entirety of the microbial genome catalogue of the cynomolgus macaque intestinal microbiome. The majority of novel SGBs belonged to the *Lachnospiraceae* family, which comprises a diversity of genera capable of metabolising dietary polysaccharides [101]. The identification of these putative novel taxa highlights the dearth of NHP-specific microbial taxa in reference databases and suggests that the cynomolgus macaque microbiome may be a source of previously unrealised microbial diversity. Future work should include functional characterisation of these SGBs to develop an understanding of their function.

### Limitations

The restricted number of animals, unequal age distribution of the cohort, and inclusion of two animals recently diagnosed with diabetes represent the main limitations of this study. The study also did not include animals > 20 years, with the typical lifespan of cynomolgus macaques in captivity being 25–30 years. It is therefore possible that the upper limit of the cohort’s age range, in addition to the size of the cohort, cannot fully capture age-associated effects on the intestinal microbiome. In addition, the limited representation of the cynomolgus macaque intestinal microbiome in commonly used reference-based profiling databases restricts characterisation of the bacterial and archaeal communities and the ability to discern age-associated alterations. In response to infection or injury, the animals within this cohort are treated with antibiotics, occasionally followed with probiotic treatment, which can influence microbial taxonomic composition [102]. This, in addition to close contact from co-housing, augmented by grooming behaviours, may collectively promote the sharing and similarities of the intestinal microbiome between individuals of different ages [39, 103–106]. Close contact between macaques of differing ages in social groups is, however, reflective of social interactions in both humans and wild cynomolgus macaques.

## Conclusions

To our knowledge, this study represents the first characterisation of both the small and large intestinal microbiome across the lifespan in captive-bred cynomolgus macaques. The distinct microbial compositions of the small intestine, caecum, and colon highlight the limitations of the ongoing and frequent use of faecal microbial community profiles as a proxy for those of the intestinal lumen. In addition, while reference-based profiling methods have improved for NHP microbiomes, allowing us to identify changes in microbial taxonomy and abundance, the age-associated alterations in abundance of a small number of putative novel taxa indicates that there may be further age and sex-associated alterations that remain undetected. There is evident need for further expansion of microbial genome reference databases to capture the full diversity of and potential age-associated changes to NHP microbiomes. The catalogue of putative novel genomes retrieved from the present dataset provides a valuable resource that contributes to this aim.

## Supplementary Information

Supplementary Material 1. Per sample read numbers following removal of reads mapping to the cynomolgus macaque genome. Per-sample read numbers for samples which retained > 1 million reads following removal of host genome reads, shown as abeeswarm plothistogramscatter plot. The correlation between age and the abundance of two putative novel bacterial species-level genome bins. SGBs were obtained from the intestinal metagenomes of captive cynomolgus macaques of differing ages. The SGBs are from theUBA11490 andRF16 genus in the proximal colon. Sample metadata. Associated metadata for each sample included within the analysis. Results of linear mixed effect models used to facilitate comparisons between alpha diversity and region. Linear mixed-effects models were used to examine the associations between region and three metrics of alpha diversityin metagenomic samples collected from different regions of the intestinal tract. Age was included as a fixed effect. Abbreviations: D = Duodenum, J = Jejunum, I = Ileum, C = Caecum, PC = Proximal Colon, DC = Distal Colon. Results of pairwise multiple comparisons following PERMANOVA. Pairwise comparisons were performed to assess differences in microbial community composition between intestinal regions and age groups. Age groups are defined as 4–7 years, 8–12 yearsand 13–20 years. Abbreviations: D = Duodenum, J = Jejunum, I = Ileum, C = Caecum, PC = Proximal Colon, DC = Distal Colon. Results of linear models used to facilitate comparisons between alpha diversity and age. Statistical results of linear models used to measure associations between three metrics of alpha diversityand age in samples from each region of the intestinal tract. Abbreviations: D = Duodenum, J = Jejunum, I = Ileum, C = Caecum, PC = Proximal Colon, DC = Distal Colon. Associations between taxonomic abundance and age, analysed using MaAsLin2. The effect of age on the differential abundance of taxa identified using reference-based computational profilingwas assessed using MaAsLin2. All results are shown here, including non-significant associations. Associations between metabolic pathway abundance and age, analysed using MaAsLin2. The effect of age on the differential abundance of MetaCyc pathways identified using HUMAnN3 was assessed using MaAsLin2. All results are shown, including non-significant associations. Putative novel species-level genome binsidentified via an assembly-based computational profiling approach. Assigned taxonomy of putative novel SGBs identified in the analysed metagenomic dataset. Associations between putative novel SGB abundance and age, analysed using MaAsLin2. The effect of age on the differential abundance of putative novel species-level genomes binsidentified using an assembly-based computational profiling approach was assessed using MaAsLin2. All results are shown, including non-significant associations. Statistical comparisons between alpha diversity and region with sex included as a fixed effect. Linear mixed-effects models were used to examine the associations between region and three metrics of alpha diversityin metagenomic samples collected from different regions of the intestinal tract. Age and sex were included as fixed effects. Abbreviations: D = Duodenum, J = Jejunum, I = Ileum, C = Caecum, PC = Proximal Colon, DC = Distal Colon. Results of pairwise multiple comparisons following PERMANOVA with sex included as a fixed effect. Pairwise comparisons were performed to assess differences in microbial community composition between intestinal regions and age groups. Sex was included as a fixed effect. Age groups are defined as 4–7 years, 8–12 yearsand 13–20 years. Abbreviations: D = Duodenum, J = Jejunum, I = Ileum, C = Caecum, PC = Proximal Colon, DC = Distal Colon. Statistical comparisons between alpha diversity and age with sex included as a fixed effect. Statistical results of linear models used to measure associations between three metrics of alpha diversityand age in samples from each region of the intestinal tract. Sex was included as a fixed effect. Abbreviations: D = Duodenum, J = Jejunum, I = Ileum, C = Caecum, PC = Proximal Colon, DC = Distal Colon. Associations between taxonomic abundance and age with sex included as a fixed effect. The effect of age on the differential abundance of taxa identified using reference-based computational profilingwas assessed using MaAsLin2. Sex was included as a fixed effect. All results are shown, including non-significant associations. Associations between metabolic pathway abundance and age with sex included as a fixed effect. The effect of age on the differential abundance of MetaCyc pathways identified using HUMAnN3 was assessed using MaAsLin2. Sex was included as a fixed effect. All results are shown, including non-significant associations. Associations between putative novel SGB abundance and age with sex included as a fixed effect. The effect of age on the differential abundance of putative novel species-level genomes binsidentified using an assembly-based computational profiling approach was assessed using MaAsLin2. Sex was included as a fixed effect. All results are shown, including non-significant associations

## Data Availability

The datasets generated and analysed during the current study are available in the NCBI Sequence Read Archive (SRA), under the project accession number PRJNA1164203 [107].

## Abbreviations

BH: Benjamini-Hochberg
CoPM: Copies per million
CPM: Counts per million
DNA: Deoxyribonucleic acid
FDR: False discovery rate
MAG: Metagenome assembled genome
NHP: Non-human primate
NMDS: Non-metric multidimensional scaling
PCR: Polymerase chain reaction
PERMANOVA: Permutational multivariate analysis of variance
rRNA: Ribosomal ribonucleic acid
SGB: Species-level genome bins
UKHSA: UK Health Security Agency
USA: United States of America
