# Supplementary figures and images for: Intestinal microbiota profiles of captive-bred cynomolgus macaques reveal influence of biogeography and age
Source: Anim Microbiome. 2025 May 14;7:47. doi: 10.1186/s42523-025-00409-9 (PMC12080069; doi:10.1186/s42523-025-00409-9)

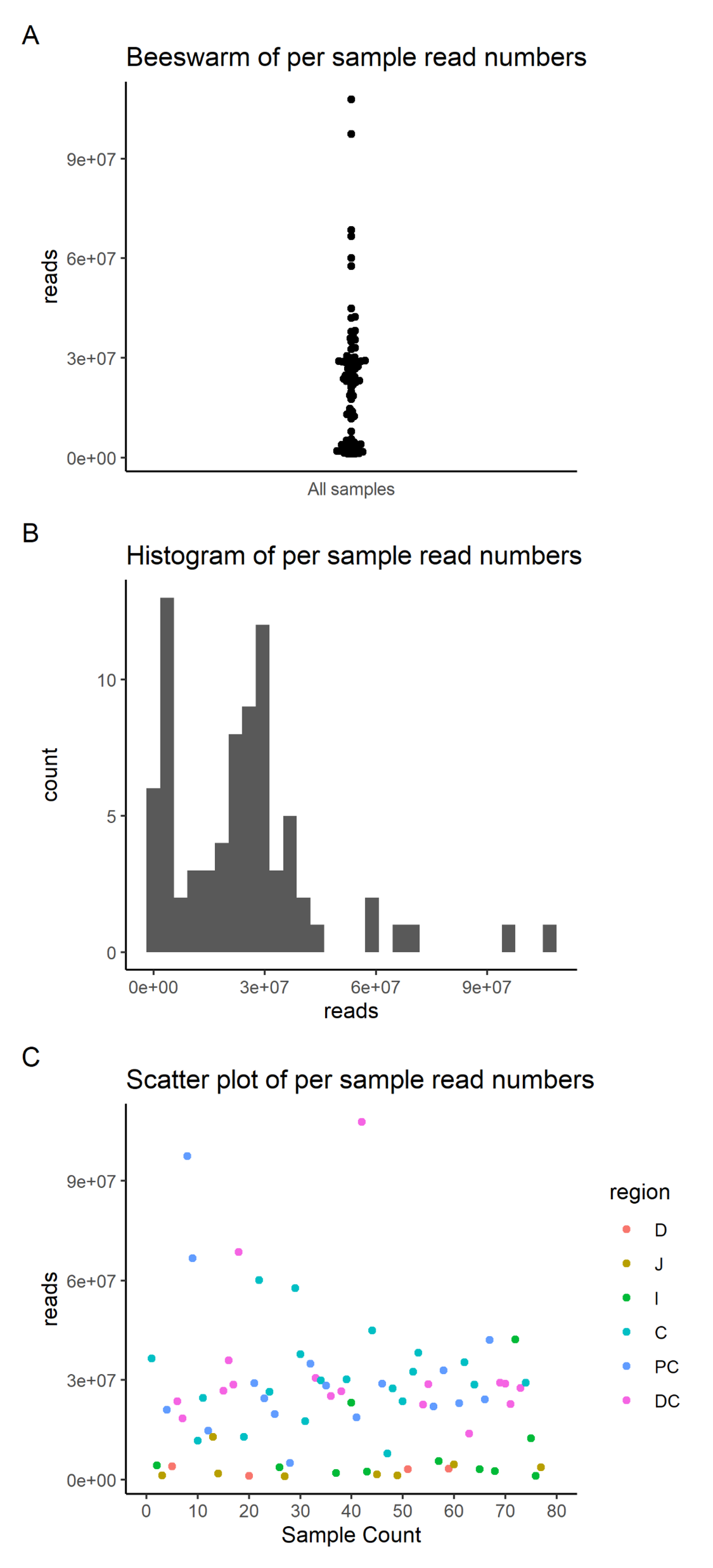

Supplement: Supplementary file 1 — Supplementary Material 1. Per sample read numbers following removal of reads mapping to the cynomolgus macaque genome. Per-sample read numbers for samples which retained > 1 million reads following removal of host genome reads, shown as abeeswarm plothistogramscatter plot. The correlation between age and the abundance of two putative novel bacterial species-level genome bins. SGBs were obtained from the intestinal metagenomes of captive cynomolgus macaques of differing ages. The SGBs are from theUBA11490 andRF16 genus in the proximal colon. Sample metadata. Associated metadata for each sample included within the analysis. Results of linear mixed effect models used to facilitate comparisons between alpha diversity and region. Linear mixed-effects models were used to examine the associations between region and three metrics of alpha diversityin metagenomic samples collected from different regions of the intestinal tract. Age was included as a fixed effect. Abbreviations: D = Duodenum, J = Jejunum, I = Ileum, C = Caecum, PC = Proximal Colon, DC = Distal Colon. Results of pairwise multiple comparisons following PERMANOVA. Pairwise comparisons were performed to assess differences in microbial community composition between intestinal regions and age groups. Age groups are defined as 4–7 years, 8–12 yearsand 13–20 years. Abbreviations: D = Duodenum, J = Jejunum, I = Ileum, C = Caecum, PC = Proximal Colon, DC = Distal Colon. Results of linear models used to facilitate comparisons between alpha diversity and age. Statistical results of linear models used to measure associations between three metrics of alpha diversityand age in samples from each region of the intestinal tract. Abbreviations: D = Duodenum, J = Jejunum, I = Ileum, C = Caecum, PC = Proximal Colon, DC = Distal Colon. Associations between taxonomic abundance and age, analysed using MaAsLin2. The effect of age on the differential abundance of taxa identified using reference-based computational profilingwas assesse [file 42523_2025_409_MOESM1_ESM.zip › Additional Files/AdditionalFile1.tif]

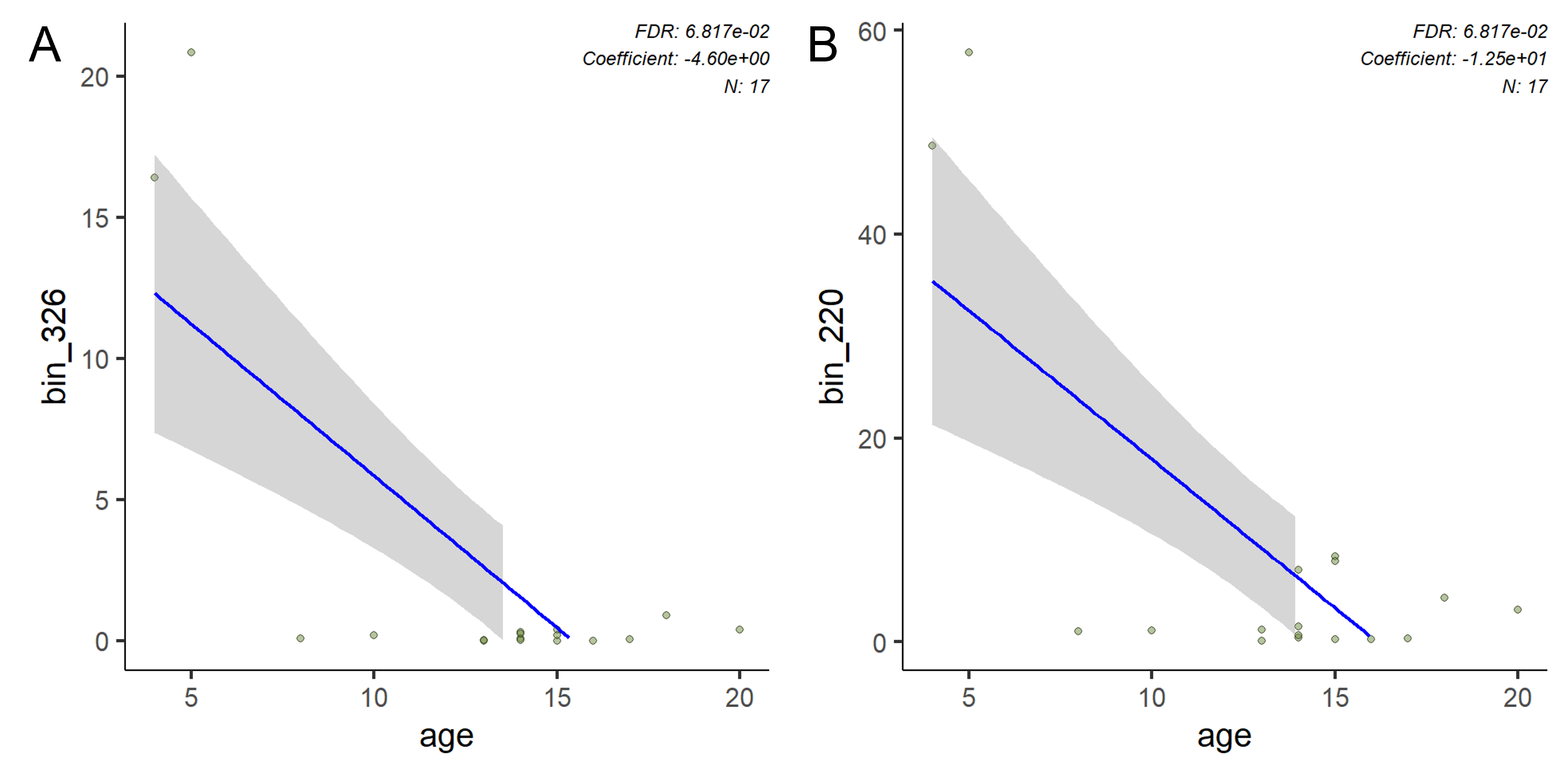

Supplement: Supplementary file 1 — Supplementary Material 1. Per sample read numbers following removal of reads mapping to the cynomolgus macaque genome. Per-sample read numbers for samples which retained > 1 million reads following removal of host genome reads, shown as abeeswarm plothistogramscatter plot. The correlation between age and the abundance of two putative novel bacterial species-level genome bins. SGBs were obtained from the intestinal metagenomes of captive cynomolgus macaques of differing ages. The SGBs are from theUBA11490 andRF16 genus in the proximal colon. Sample metadata. Associated metadata for each sample included within the analysis. Results of linear mixed effect models used to facilitate comparisons between alpha diversity and region. Linear mixed-effects models were used to examine the associations between region and three metrics of alpha diversityin metagenomic samples collected from different regions of the intestinal tract. Age was included as a fixed effect. Abbreviations: D = Duodenum, J = Jejunum, I = Ileum, C = Caecum, PC = Proximal Colon, DC = Distal Colon. Results of pairwise multiple comparisons following PERMANOVA. Pairwise comparisons were performed to assess differences in microbial community composition between intestinal regions and age groups. Age groups are defined as 4–7 years, 8–12 yearsand 13–20 years. Abbreviations: D = Duodenum, J = Jejunum, I = Ileum, C = Caecum, PC = Proximal Colon, DC = Distal Colon. Results of linear models used to facilitate comparisons between alpha diversity and age. Statistical results of linear models used to measure associations between three metrics of alpha diversityand age in samples from each region of the intestinal tract. Abbreviations: D = Duodenum, J = Jejunum, I = Ileum, C = Caecum, PC = Proximal Colon, DC = Distal Colon. Associations between taxonomic abundance and age, analysed using MaAsLin2. The effect of age on the differential abundance of taxa identified using reference-based computational profilingwas assesse [file 42523_2025_409_MOESM1_ESM.zip › Additional Files/AdditionalFile2.tif]
